# Validation of the German version of two scales (RIS, RCS-HCP) for measuring regret associated with providing healthcare

**DOI:** 10.1186/s12955-017-0630-z

**Published:** 2017-03-24

**Authors:** Silvia C. Richner, Stéphane Cullati, Boris Cheval, Ralph E. Schmidt, Pierre Chopard, Christoph A. Meier, Delphine S. Courvoisier

**Affiliations:** 1 Department of Internal Medicine and Specialties, Stadtspital Triemli, Zurich, Switzerland; 2 Geneva University Hospitals, Geneva, Switzerland; 3 University of Geneva, Geneva, Switzerland; 4 Office of the Chief Medical Officer, University Hospital Basel, Basel, Switzerland

**Keywords:** Regret, Validation, Translation, Healthcare professionals, Psychometrics, Switzerland, Days of work absence

## Abstract

**Background:**

The regret intensity scale (RIS) and the regret coping scale for healthcare professionals (RCS-HCP) working in hospitals assess the experience of care-related regrets and how healthcare professional deal with these negative events. The aim of this study was to validate a German version of the RIS and the RCS-HCP.

**Methods:**

The RIS and RCS-HCP in German were first translated into German (forward- and backward translations) and then pretested with 16 German-speaking healthcare professionals. Finally, two surveys (test and 1-month retest) administered the scales to a large sample of healthcare professionals from two different hospitals.

**Results:**

Of the 2142 eligible healthcare professionals, 494 (23.1%) individuals (108 physicians) completed the cross-sectional web based survey and 244 completed the retest questionnaire. Participants (*n* = 165, 33.4% of the total sample) who reported not having experienced a regret in the last 5 years, had significantly more days of sick leave during the last 6 months. These participants were excluded from the subsequent analyses. The structure of the scales was similar to the French version with a single dimension for the regret intensity scale (Cronbach’s alpha: 0.88) and three types of coping strategies for the regret coping scale (alphas: 0.69 for problem-focused strategies, 0.67 for adaptive strategies and 0.86 for the maladaptive strategies). Construct validity was good and reproduced the findings of the French study, namely that higher regret intensity was associated with situations that entailed more consequences for the patients. Furthermore, higher regret intensity and more frequent use of maladaptive strategies were associated with more sleep difficulties and less work satisfaction.

**Conclusions:**

The German RIS and RCS-HCP scales were found valid for measuring regret intensity and regret coping in a population of healthcare professionals working in a hospital. Reporting no regret, which corresponds to the coping strategy of suppression, seems to be a maladaptive strategy because it was associated with more frequent sick day leaves.

**Electronic supplementary material:**

The online version of this article (doi:10.1186/s12955-017-0630-z) contains supplementary material, which is available to authorized users.

## Background

Healthcare professionals are increasingly providing care to complex patients of older age, with multiple comorbidities [1] and from various cultural origins. How healthcare professionals respond to disparate groups of patients is a challenge, as well as how they deal with the contradictory needs of being empathic clinicians and dealing with the emotional burden of patients’ suffering, complications, or death [2]. Providing patient-centered and family-focused care implies more challenging medical and clinical decisions and the risk for unsatisfactory patient outcomes is greater, thereby generating strong negative emotions among healthcare professionals, such as ‘stress of consciences’ [3], moral distress [4], or feelings of loss of control [5].

Regret is a frequent emotional experience, which may be defined as a psychological state following an experience where one believes that the outcome would have been better if one had acted differently [6]. Regret develops in situations where healthcare professionals cannot fulfill what they believe to be the optimal care for their patients. In a cross-sectional survey of healthcare professionals in Switzerland, the prevalence of regret in a one-month period was 15% among nurses and 10% among physicians [7]. To cope with feelings of regret, people may use various strategies: the main distinction among coping strategies is problem-focused versus emotion-focused [8, 9]. Problem-focused coping strategies are directed towards reducing or eliminating a stressor or solving the situation, whereas emotion-focused coping strategies are directed towards changing one's own emotional reaction to the situation.

Regret in the healthcare setting occurs mainly when clinicians perceive their care as inappropriate [10] or futile [11], and in the context of defensive medicine [12]. Regrets also occur when clinicians are implicated in patient-adverse events or medical errors [13]. “Second victim” experiences are closely related to regret feelings [14, 15]. The consequences of these strong feelings manifest themselves at different levels. At the individual level, regret can lead to sleep problems [16–20], which can result in concentration deficits and higher risk of errors [21] or contribute to burnout [22]. At the patient-care level, regret can affect decision making [23], as well as learning and changing practice for future interventions [24–26], and again is associated with higher risk of error [16]. The decision-making process, especially in situations associated with a high workload, can trigger a variety of emotional reactions [27]. For example, anticipated regret is known to play a substantial role when physicians favor action (e.g., additional diagnostic tests) instead of inaction [24, 28]. At the institutional level, distress may increase turnover of staff members and days of sick leaves [29, 30].

Healthcare professionals’ emotional reactions have increasingly attracted scientific attention over the last decade [31–33]. However, there is a lack of valid instruments to measure regret intensity and regret regulation strategies in healthcare professionals [34], although regret can be conceptually quantified by the intensity of the emotion and coping can be assessed by the frequency of use of the various strategies [13, 35]. Such instruments should cover the main dimensions of emotion and emotion regulation, and should be reliable yet short, in order to allow monitoring regret at regular intervals. Two French speaking scales measuring regret intensity [36] and coping strategies [7] fulfill these requirements. Thus, the aim of this study is to translate and validate a German version of the Regret Intensity Scale (RIS) and the Regret Coping Scale of Health Care Professionals (RCS-HCP) [7, 36], developed in Switzerland.

## Methods

### Design

The steps to validate the RIS and RCS-HCP in German were first to contact two healthcare professionals in the German-speaking part of Switzerland (one physician, one psychotherapist) and to assess the conceptual validity of the scales in their cultural context. The scales were then translated, and pretested among German-speaking healthcare professionals. The final step was to use two surveys (test and retest) to administer the scales to a large sample of healthcare professionals from two different hospitals. The Ethics Committee of Zurich indicated that the research was exempted from formal research ethics approval because, as declared by Swiss law, it did not study a disease and did not use an intervention on health.

### Scale translation

Two professional translators with expertise in the field of healthcare independently performed the translation of the two scales and the validation questions from French to German (forward translation). Then, two native French translators independently translated the German version back into French (backward translation). The backward translators were unaware of the original scales in French. A group of experts including three native German speakers and three native French speakers (two psychologists, three physicians and one medical sociologist) examined the translated items and selected the best translation for each item after the forward translation and after the backward translation.

### Pretest

The first German versions of the Regret Intensity Scale (RIS) and Regret Regulation Scale (RCS-HCP) were pretested using one-on-one structured interviews by one interviewer at Stadtspital Triemli among 7 nurses, 8 doctors and 1 psychologist from a variety of clinics. The objective was to ensure that the translated items were clear and understandable for different professionals. During this process, 3 successive small adaptations and new versions of the questionnaire were made (see Additional file 1 for the final versions of these scales). There were no suggestions about additional domains that should be assessed.

### Participants of the main survey

All professional email addresses of nurses and physicians of the Stadtspital Triemli, Zurich (500 bed hospital) and the Bezirksspital Affoltern am Albis (100 bed hospital) were collected. The participants, coming from all different departments and clinics, were informed by posters and flyers one week before the email was sent. Participants were informed that a small incentive for each completed questionnaire will be donated to the foundation Theodora (Giggle doctors for children: http://ch.theodora.org). The inclusion criteria were healthcare professionals currently working with patients; the exclusion criteria were professionals not having worked with patients for at least 5 years or retired.

### Sample size calculation

To determine sample size, we used the rule of 10 respondents per 1 item [37] A subject to item ratio of 10 was an adequate compromise between goodness quality of factor analysis estimation (supposing large samples), the low and declining participation rates of healthcare professionals in surveys [23] and the small size of the two hospitals where the survey was conducted. Considering that the longest scale (RCS-HCP) has 15 items, 150 respondents were required. In order to be able to examine the psychometric properties of the scales separately among nurses and physicians, the minimum sample size was fixed at 300 (150 physicians, 150 other professionals).

### Procedure

After the pretest of the translated questionnaire a cross-sectional survey with a web questionnaire was conducted. Up to three reminders were sent to the professional email addresses at a one-week interval. One month later the same questionnaire was sent to the participants who accepted to receive the retest.

### Measurements

The questionnaire sent to all the participants contained, after an introduction and clear definition of the term regret, a single question about the most important regret, 6 questions about the consequences for the patient of the regretted situation (whether the regretted situation led to death, longer hospital stay, transfer to intensive care unit (ICU), extra surveillance, reanimation measures or durable physical or psychological handicap). There was also a single question about how much the respondent felt responsible for the situation (visual analogue scale from 0, Not at all, to 10, Very Responsible) and a question about whether the respondent felt this situation was an error (yes vs no). Regret intensity was measured by the German version of the regret intensity scale (RIS; 10 statements rated from strongly disagree (1) to strongly agree (5), which showed a good reliability with a Cronbach’s alpha = 0.87 in the French version). Regret coping strategies were measured by the German version of the regret coping scale for healthcare professionals [RCS-HCP; 15 statements rated from never or almost never (1) to always or almost always (4)]. This scale examines three types of coping strategies: problem-focused, maladaptive emotion-focused (self-attacking and rumination), and adaptive emotion-focused (all other strategies, considered as potentially helpful). All subscales measuring coping strategies showed good reliability in the French version, respectively 0.89, 0.89, and 0.89.

To assess construct validity, the insomnia severity index, the general job satisfaction scale and a general self-rated health question were added. The insomnia severity index (ISI) consists of 7 items rated from 0 to 4 (total score range: 0–28), with a higher score indicating more insomnia symptoms, with good alpha = 0.80. In a community sample, a threshold at 10 discriminated well between people with and without insomnia (as evaluated by a clinical interview) and the minimal important difference was 1.5 [38]. The general job satisfaction scale consists of 5 items on a 7-point scale [score ranging from Low (1) to High (7)] with a relatively low Cronbach’s alpha 0.61 [39]. The general self-rated health question corresponds to the first question from the SF-36 questionnaire, and has good criterion validity as it predicts mortality [40]. At the end of the survey, information on the socio-demographic and professional status of the participants were collected.

### Statistical analyses

Participants who reported not having experienced a regret in the last 5 years were excluded from the analysis*.* Analyses related to the structure of the questionnaires were first run separately for physicians and nurses. Because the results were similar, analyses were then reported for the whole sample. For each item of the RIS and RCS-HCP, the percentage of the lowest and highest value was described. For each scale, we used principal component analysis to examine the number of underlying dimensions of the scale. If the scale had more than one component, we used exploratory factor analysis to obtain factor loadings and determine which items belong to which subscales. Analyses using item response theory for polytomous items (graded response model) supported the results of the factor analyses. The structure of the scales was also confirmed using confirmatory factor analysis, and reporting the recommended goodness-of-fit criteria and threshold: Chisquare/degrees of freedom (*χ* ^2^/df) should be <3, RMSEA should be <0.8, SRMR should be <0.10, and CFI should be >0.95 [41]. For each subscale, reliability was estimated using Cronbach’s alpha. Test-retest reliability was estimated using weighted kappa for items and intra-class correlation (ICC2) for total scores. In addition, for the RIS and the three coping strategies scores, their variability over time (i.e., measurement error) was examined by a Bland-Altman plot of the participants means at baseline and 1-month follow-up versus the differences in the scores [42]. The agreement interval of the differences provides the limits of agreement. At the end, construct validity was examined using correlations between continuous variables and t-tests when the construct validity variables were dichotomous. Analyses were done using R v3.3.1 (R foundation, Vienna, Austria).

## Results

### Sample characteristics

Of 2196 participants who received an email invitation, 54 were excluded because they reported not working with patients for the last five years or being out of work, a further 148 refused to participate, and 1500 did not answer. Of the remaining 494 (23.1%) participants, 369 (74.7%) agreed to be contacted after one month, and 244 (66.1%) completed the questionnaire a second time.

The mean age of the participants was 39.1 years (SD = 10.2). The majority were women (81.8%), around one fourth of the respondents were physicians (21.9%). Professions other than medical doctors were grouped together for simplicity and because very few participants had a profession other than nurse or physician. Most employees worked at 80% or more. With respect to their health status, more than 25% of the healthcare professionals reported at least one sick leave day in the last 6 months, and 5% of the participants considered their health status as fair or poor. Healthcare professionals reported average levels of regret intensity (mean = 2.04, SD = 0.78, range =1-5) under the scale midpoints. For the coping strategies (range 1–4) they reported average levels of adaptive strategies (mean = 2.59, SD = 0.57) and problem-focused strategies (mean = 2.83, SD = 0.61) above the scale midpoints. Conversely, maladaptive strategies (mean = 1.78, SD = 0.63) were slightly under the scale midpoints.

Of the 494 participants, 165 (33.4%) reported not having experienced a regret in the last 5 years (Table 1). When compared with the participants reporting regrets, those reporting no regret were similar in terms of demographic characteristics (age, gender), as well as in job characteristics (clinical activity percentage, supervisor status and night-shift load). However, a significantly larger proportion of participants reporting no regret were non-physicians, and had between 6 and 10 years of experience. Furthermore, respondents reporting no regret indicated more often having had >3 days of sick leave during the last 6 months. This increased proportion of persons with a sick leave >3 days among healthcare professionals reporting no regret was similar for physicians and for non-physicians, though the smaller sample size did not allow for significant associations within professions. Indeed, among physicians, the proportion of sick leave was 1.1% when the reported at least one regret and 10.5% when they reported no regret (*p* = 0.06). Among non-physicians, these proportions were 6.0% versus 10.1%, respectively (*p* = 0.16).

**Table 1:** Comparison of healthcare professionals’ characteristics between participants who reported at least one regret and participants who reported having never had a regret during the 5 last years

		No regret *N* = 165	At least 1 regret *N* = 329	*P*-value
Age	<30	34 (21.7%)	56 (18.1%)	0.24
	31–39	61 (38.9%)	101 (32.7%)	
	40–49	39 (24.8%)	90 (29.1%)	
	>50	23 (14.6%)	62 (20.1%)	
Sex	Woman	138 (83.6%)	266 (80.9%)	0.52
	Man	27 (16.4%)	63 (19.1%)	
Profession	Non Physicians	138 (87.9%)	217 (70.9%)	<0.001
	Physicians	19 (12.1%)	89 (29.1%)	
Professional status	Nurse/resident	115 (74.2%)	201(66.8%)	0.13
	Supervisor	40 (25.8%)	100 (33.2%)	
Percentage of clinical activity	0–50%	14 (9.0%)	22 (7.1%)	0.76
	51–80%	41 (26.3%)	85 (27.4%)	
	81–100%	101 (64.7%)	203 (65.5%)	
Years of Experience	1–2	8 (5.2%)	24 (7.9%)	0.002
	3–5	14 (9.1%)	41 (13.5%)	
	6–10	39 (25.3%)	33 (10.9%)	
	11–20	46 (29.9%)	109 (36.0%)	
	>20	47 (30.5%)	96 (31.7%)	
Nightshifts during last month	0	75 (48.1%)	143 (45.8%)	0.52
	1–3	26 (16.7%)	48 (15.4%)	
	4–6	36 (23.1%)	63 (20.2%)	
	7–9	13 (8.3%)	39 (12.5%)	
	>9	6 (3.8%)	19 (6.1%)	
Self-reported health	Excellent	25 (15.2%)	63 (19.1%)	0.66
	Very good	70 (42.4%)	132 (40.1%)	
	Good	54 (32.7%)	95 (28.8%)	
	Fair	10 (6.1%)	18 (5.5%)	
	Poor	0	1 (0.3%)	
Sick leave during last 6 month	None −3 days	143 (89.4%)	295 (94.9%)	0.04
	>3 days	17 (10.6%)	16 (5.1.%)	
Scores, mean (SD)				
Job Satisfaction (range 1–7)		4.8 (1.1)	4.7 (1.1)	0.16
Sleep (range 0–28)		6.4 (4.9)	5.9 (4.9)	0.25

*SD* standard deviation

### Internal validity

Similarly to the French version, the principal component analysis of the regret intensity scale found a single component with all loadings above 0.40 (Table 2). The item with the lowest loading was “I feel anger rising in me” (“steigt Wut in mir auf.”). Confirmatory factor analysis showed a good fit of the model to the data: *χ* ^2^/df = 2.3, RMSEA = 0.07 (95% confidence interval: 0.06–0.08), SRMR = 0.07, CFI = 0.95. The Cronbach’s alpha was 0.88. The RIS, and the three RCS-HCP scores did not show any floor or ceiling effect.

**Table 2:** Item characteristics of regret intensity at first survey (*n* = 329). Number left of item description corresponds to the item order in the scale

When I think about the situation that caused my major regret…	% at lowest value	% at highest value	Mean (SD)	Loading of PCA
RIS-10				
1. Emotions come back to me	8.0	18.7	3.14 (1.22)	0.63
2. I feel uncomfortable	14.7	12.2	2.8 (1.23)	0.74
3. I feel devalued	52.8	4.1	1.88 (1.16)	0.77
4. I feel ashamed	44.4	7.8	2.17 (1.31)	0.73
5. I have a knot in my stomach	43.0	6.5	2.19 (1.28)	0.75
6. I feel anger rising in me	43.6	8.5	2.23 (1.36)	0.46
7. At home, I have trouble falling asleep	70.0	1.6	1.56 (1.0)	0.62
8. At work, I have trouble concentrating	74.6	0.3	1.35 (0.70)	0.68
9. I have the impression I am not really made for this work anymore	68.0	3.4	1.60 (1.06)	0.60
10. I want to cry	71.7	0.9	1.45 (0.83)	0.68
10-item regret intensity scale	2.2	0.0	2.0 (0.78)	–

*SD* standard deviation, *PCA* Principal Component Analysis

With respect to the regret coping scale, the principal component analysis suggested 3 components based on Kaiser’s criterion and the screeplot. The loadings of the 3-factor structure reproduced the structure of the French version of the scales, with 5 items measuring problem-focused strategies, 5 items measuring emotion-focused strategies that are mostly maladaptive, and 5 items measuring adaptive emotion-focused strategies (Table 3). Confirmatory factor analysis showed an acceptable fit of the model to the data: *χ* ^2^/df = 2.1, RMSEA = 0.06 (95% confidence interval: 0.05–0.07), SRMR = 0.06, CFI = 0.96. The Cronbach’s alpha was 0.69 for problem-focused, 0.86 for maladaptive, and 0.67 for adaptive strategies.

**Table 3:** Item characteristics of regret coping at first survey (*n* = 329). Number left of item description corresponds to the item order in the scale

In general, when I regret events or situations with patients….	% at lowest value	% at highest value	Mean (SD)	Loading of FA		
				PF	MA	A
RCS-HCP						
1. I talk about it with colleagues, to be listened to or reassured	2.5	34.6	3.04 (0.84)	0.51		
2. I discuss the problem again with the patient (or his family)	22.8	12.7	2.26 (0.95)	0.51		
3. I try to find concrete solutions to the situation	1.94	54.4	3.38 (0.77)	0.50		
4. I talk with a supervisor to prevent these events from recurring	10.1	27.0	2.74 (0.97)	0.63		−0.11
5. I try to accept the situation	4.8	28.3	2.94 (0.85)	0.11		0.49
6. I think I am no good	41.6	2.6	1.77 (0.78)	−0.12	0.55	
7. I turn these situations in my head all the time	38.3	4.2	1.84 (0.81)		0.85	
8. I think about it so much that it becomes invasive	61.1	2.6	1.52 (0.75)		0.79	
9. I have a tendency to blame myself	17.0	10.2	2.24 (0.85)		0.70	
10. I tell myself that error is human	13.7	11.5	2.41 (0.86)			0.63
11. I try to take some emotional distance	4.5	23.0	2.84 (0.83)			0.54
12. I think about this situations all the time	56.9	2.9	1.55 (0.74)		0.82	
13. I expose this situation to colleagues to improve our practices	9.0	23.1	2.74 (0.92)	0.71		
14. I try to see the positive side of things	9.7	17.2	2.60 (0.88)	0.17	0.17	0.60
15. I try to put the situation in perspective	24.5	6.5	2.15 (0.87)			0.48
Problem focused	0.3	1.9	2.83 (0.61)	–	–	–
Maladaptive	8.3	0.6	1.78 (0.63)	–	–	–
Adaptive	0.3	2.6	2.59 (0.57)	–	–	–

*SD* standard deviation, *PCA* Principal Component Analysis, *FA* factor analysis, *PF* problem focused, *MA* maladaptive, *A* adaptive

### Construct validity

Table 4 presents the association of regret intensity with the consequences of the regret-inducing event. Perceived regret intensity was associated with the consequences for the patient and with patients’ unexpected death or death earlier than expected. Furthermore, regret intensity was higher when healthcare professionals felt more responsible for the situation. It was also associated with lower job satisfaction, more sleep problems and lower self-reported health.

**Table 4:** Association of regret intensity with the consequences of the regret-inducing situation on the patient, on the healthcare professional and on patient care

	Scale range	Mean difference	*p*
Consequence to patient (e.g., transfer to ICU, resuscitation, permanent harm)	1–6	0.24	0.005
Death (unexpected, or expected but earlier)	yes vs. no	0.26	0.04
Involvement of the healthcare professional			
Self-reported error	yes vs. no	0.21	0.02
		Correlation	*p*
Responsibility	0–10	0.24	<0.001
Consequences to the healthcare professional			
Satisfaction with work	1–7	−0.27	<0.001
Sleep difficulties	1–28	0.27	<0.001
Self-reported health	1–5	−0.17	0.004

Mean difference for categorical variables and Correlation for continuous variables

With respect to regret coping strategies, problem-focused strategies were more frequent among supervisors (mean difference: 0.23, *p* = 0.002). Table 5 shows the association of regret coping strategies with healthcare professionals’ characteristics. There was a positive association between the intensity of the most regretted situation and the use of maladaptive strategies. Satisfaction with work was positively associated with the use of problem-focused and of adaptive strategies and negatively with the use of maladaptive strategies. Sleep problems were associated with more frequent use of maladaptive strategies, and, marginally, with less frequent use of problem-focused strategies. Finally, self-reported health was better among healthcare professionals who more frequently used adaptive strategies.

**Table 5:** Association of regret coping strategy with the consequences of the regret-inducing situation on the healthcare professional and their characteristics

	Problem-focused		Maladaptive		Adaptive	
	Corr	*p*	Corr	*p*	Corr	*p*
RIS-10	−0.01	0.86	0.53	<0.001	−0.09	0.10
Satisfaction with work	0.24	<0.001	−0.24	<0.001	0.17	0.003
Sleep difficulties	−0.12	0.06	0.25	<0.001	−0.06	0.32
Self-reported health	0.03	0.61	−0.03	0.58	0.13	0.03

*RIS-10* regret intensity scale of most regretted situation

### Test-retest reliability

For the test-retest results of the regret intensity scale, the participants who referred to a different regret-inducing event between the test and the retest were excluded. The intensity scale and the coping scale showed similar values across the two surveys with ICC ranging between 0.36 and 0.50 for the items of the RIS, and 0.36 and 0.63 for the items of the 3 coping subscales. The ICC of the overall scales were 0.52 for the RIS, and 0.68, 0.72, 0.60 for the problem-focused (PF), maladaptive (MA) and adaptive scales (A), respectively.

**Fig. 1:**
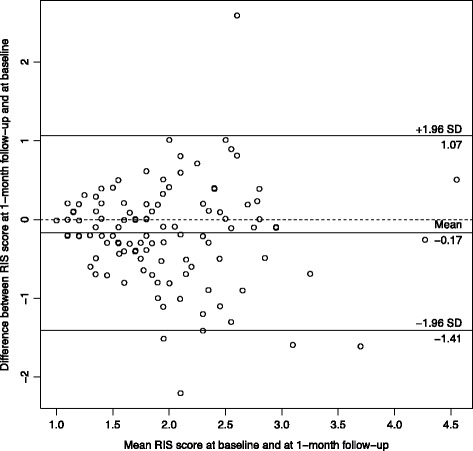
Bland-Altman plot of regret intensity (RIS-10) for the baseline and the 1‐month follow-up survey

**Fig. 2:**
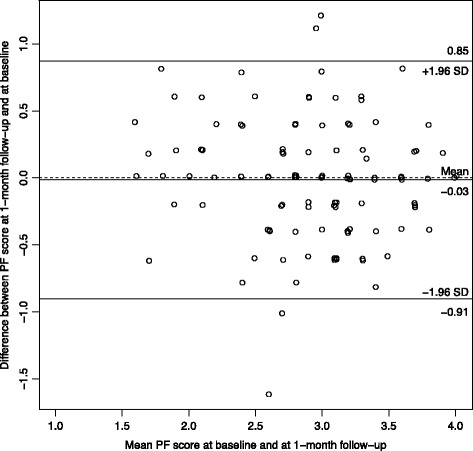
Bland‐Altman Plot of problem focusing coping strategies RCS-HCP 15 for the baseline and the 1‐month follow-up survey

**Fig. 3:**
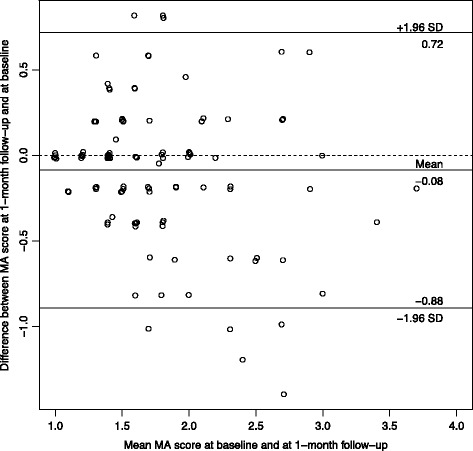
Bland‐Altman plot of maladaptative Coping strategies RCS‐HCP 15 for the baseline and the 1‐month follow‐up survey

**Fig. 4:**
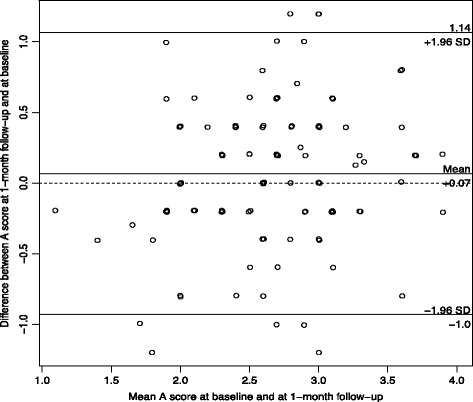
Bland‐Altman plot of adaptative coping strategies RCS-HCP 15 for the baseline and the 1‐month follow-up survey

The Bland-Altman figures (Figs. 1, 2, 3 and 4) for the baseline and the 1-month follow-up for regret intensity and the 3 regret coping strategies showed a good stability over time, with only a few healthcare professionals having a large change between baseline and follow-up, irrespective of the initial level.

## Discussion

This study examined the reliability and validity of the German versions of the RIS and RCS-HCP. These instruments were found valid for measuring regret intensity and regret coping in a population of physicians and other healthcare professionals working in a hospital.

With respect to the reliability of the instruments, analogous factor structures were obtained with the French and the German versions of the scales. Furthermore, the Cronbach’s alpha of the RIS was good and almost identical to that of the French version (0.88 in this study compared to 0.89 in the French version). However, two dimensions of the regret coping scale had lower Cronbach’s alpha at 0.69 for problem-focused strategies and 0.67 for adaptive coping. This decrease could be due to differences in language and culture, but could also be due to differences in hospital type, because the two German-speaking hospitals included in this study were not teaching hospitals. Regarding linguistic and cultural differences, there were two important differences between the French and the German versions. One concerned the word ‘regret’. In the pretest interviews and in the comments on the survey, the term ‘Gefühl des Bereuens’ (i.e., our translation of regret) was criticized by the respondents as being a word they do not use very often. The German semantics has a more moralizing and judgmental connotation than the English or French word ‘regret’. The other difference concerned the question ‘I feel anger rising in me’, which had a substantially worse loading in the German translation. A hypothesis could be that the valence of anger in the German word ‘Wut’ is more intense or that in German-speaking regions the word ‘Wut’ is less acceptable [43] than the French word ‘colère’.

With respect to construct validity, results were again quite similar to those obtained with the French instruments, showing associations of regret intensity and regret coping with sleep problems, work satisfaction, and self-reported health. Thus, this study provides additional evidence indicating that the experience of intense regrets is associated with poor work satisfaction and more sleeping problems, which could be one reason for the high turnover in healthcare professions [22, 44]. However, the magnitudes of the associations were lower than those found with the French version of the scales, certainly in part due to the lower reliability of the German scales [45].

In addition to the validation of the scales, two results should be noted. First, as in the Geneva sample, about one third of the respondents did not report any regret. Yet, regret is a frequent emotion and is a common experience in healthy individuals [46]. Thus, reporting no regret seems unrealistic, and can be considered as a coping strategy. In this study, however, we found that this strategy (which can be seen as an extreme form of suppression, namely denial) was associated with more sick-day leaves, and, albeit non-significantly, with more sleep problems [17, 20]. This suggests that denial of regrets is a maladaptive strategy of coping with this experience, though it is possible that other factors, such as motivation towards their job, may also influence reporting no regret and health outcomes. A second interesting point was that with increasing regret intensity, people are more likely to use maladaptive coping strategies. This finding suggests that using ineffective coping strategies may happen when the situation experienced exceeds the healthcare professionals’ coping abilities. Thus, providing training in regret coping but also providing support for healthcare professionals who experienced intense distress is of paramount importance for healthcare professionals’ health, job performance, and quality of life.

There are several limitations to this study. First, it is a cross-sectional study, which only allows the estimation of associations but cannot show causality. Second, the response rate was low (23.1%), in line with many Internet surveys [47]. Reasons for the low response rate could be the fact that the participants were contacted via their professional email addresses briefly; some professionals were absent during the whole study time (vacation, maternity leave). Another hypothesis is that talking about regrets and emotions in general is a delicate topic for healthcare professionals. While the low response rate should not influence the validation of the scales, it questions the representativeness of the sample and may bias, for instance, the estimation of the prevalence of intense regret. In the same vein, the sample of our study mainly involved women and healthcare professionals other than physicians, and generalizability may thus be compromised. Thus, further studies should aim to obtain a representative sample using other methodologies. Finally, our study validated the instruments in a sample of healthcare professionals who admitted having experienced work-related regret. Since a third of the sample did not admit to feeling regret, a self-report measure cannot assess the intensity of their emotion following a potentially difficult situation. For these healthcare professionals, alternative measures able to detect processes either inaccessible to introspection or that the person might want to conceal may be necessary. Such measures include objective physiological manifestation of distress [48] or implicit measurement procedure [49] based on reaction time to assess automatic associations between regret and work.

## Conclusions

The German version of the RIS and RCS- HCP are valid and reliable instruments to assess regret intensity and the use of coping strategies among healthcare professionals working in hospitals. Reporting no regret, which corresponds to the coping strategy of suppression, seems to be a maladaptive strategy because it was associated with more frequent sick day leaves. A practical implication of our study is that it may help evaluate important aspects of emotion regulation that frequently occur in healthcare professional settings. Further studies are needed to develop interventions specifically designed to help healthcare professionals to deal with emotionally challenging work experiences [50, 51].

## Additional file

Additional file 1: German version of the RIS and RCS-HCP scales. (DOCX 61 kb)

## Abbreviations

A: Adaptive
CFI: Comparative fit index
df: Degree of freedom
ICC: Intra-class correlation
ICU: Intensive care unit
ISI: Insomnia severity index
MA: Maladaptive
PF: Problem-focused
RCS-HCP: Regret coping scale for healthcare professionals
RIS: Regret intensity scale
RMSEA: Root mean squared error of approximation
SF-36: The short form (36) health survey
SRMR: Standardized root mean residual
