# Erratum to: Validation of the German version of two scales (RIS, RCS-HCP) for measuring regret associated with providing healthcare

**DOI:** 10.1186/s12955-017-0653-5

**Published:** 2017-04-12

**Authors:** Silvia C. Richner, Stéphane Cullati, Boris Cheval, Ralph E. Schmidt, Pierre Chopard, Christoph A. Meier, Delphine S. Courvoisier

**Affiliations:** 1 Department of Internal Medicine and Specialties, Stadtspital Triemli, Zurich, Switzerland; 2 Geneva University Hospitals, Geneva, Switzerland; 3 University of Geneva, Geneva, Switzerland; 4 Office of the Chief Medical Officer, University Hospital Basel, Basel, Switzerland

## Erratum

The authors of the original article [1] mistakenly omitted information in the Funding section of the Declarations.

As such, the authors would like to acknowledge that the study was supported by a grant from the Swiss National Fund (100019_166010).
