# Supplementary material for: Validation of the German version of two scales (RIS, RCS-HCP) for measuring regret associated with providing healthcare
Source: Health Qual Life Outcomes. 2017 Mar 24;15:56. doi: 10.1186/s12955-017-0630-z (PMC5364621; doi:10.1186/s12955-017-0630-z)
Supplement: Additional file 1: — German version of the RIS and RCS-HCP scales. (DOCX 61 kb) [file 12955_2017_630_MOESM1_ESM.docx]

**Additional files:**

RIS-10

In welchem Maß treffen die folgenden Aussagen ***heute*** auf Sie zu, wenn Sie an diese bereute Situation zurückdenken? *(in jeder Zeile die passende Antwort ankreuzen)*

überhaupt auf

nicht jeden Fall

***Wenn ich an die Situation zurückdenke,***

***die ich am meisten bereue,...***

1. kommen Emotionen in mir wieder hoch 1 2 3 4 5
2. fühle ich mich unwohl in meiner Haut 1 2 3 4 5
3. fühle ich mich weniger wert 1 2 3 4 5
4. schäme ich mich 1 2 3 4 5
5. spüre ich einen Knoten im Magen 1 2 3 4 5
6. steigt Wut in mir auf 1 2 3 4 5
7. habe ich zu Hause Schwierigkeiten einzuschlafen 1 2 3 4 5
8. kann ich mich bei der Arbeit nur schwer konzentrieren. 1 2 3 4 5
9. habe ich den Eindruck, nicht mehr wirklich für

meinen Beruf geschaffen zu sein. 1 2 3 4 5

1. möchte ich am liebsten weinen 1 2 3 4 5

RCS-HCP 15

Geben Sie nun ganz allgemein an, wie häufig die folgenden Aussagen auf **Ihr Erleben zutreffen** (*in jeder Zeile bitte die passende Antwort ankreuzen*)

, immer oder

fast immer

oft

manchmal

***Im Allgemeinen wenn ich Ereignisse oder***

***Situationen mit Patienten bereue, ...*** nie oder

fast nie

1. dann spreche ich mit Kollegen, damit mir jemand

zuhört oder mich bestärkt 1 2 3 4

1. bespreche ich das Problem nochmals mit dem Patienten

(oder seiner Familie) 1 2 3 4

1. bemühe ich mich, konkrete Lösungen für die Situationen

zu finden 1 2 3 4

1. spreche ich mit einem Kader, um zu verhindern,

dass sich solche Situationen wiederholen 1 2 3 4

1. versuche ich, die Situation zu akzeptieren 1 2 3 4
2. fühle ich mich unfähig 1 2 3 4
3. drehen sich meine Gedanken ununterbrochen um diese

Situationen 1 2 3 4

1. denke ich so oft daran, dass es mich vereinnahmt 1 2 3 4
2. neige ich dazu, mir Vorwürfe zu machen 1 2 3 4
3. sage ich mir, dass es menschlich ist, Fehler zu machen 1 2 3 4
4. versuche ich, Abstand zu gewinnen 1 2 3 4
5. muss ich pausenlos an diese Situationen denken 1 2 3 4
6. lege ich die Situation meinen Kollegen dar, um unsere

Arbeitsweise zu verbessern 1 2 3 4

1. bemühe ich mich, die Dinge von der positiven Seite zu

sehen 1 2 3 4

1. versuche ich, die Situation zu relativieren 1 2 3 4
